# Association between serum lipid levels, osteoprotegerin and depressive symptomatology in psychotic disorders

**DOI:** 10.1007/s00406-018-0897-z

**Published:** 2018-05-02

**Authors:** Sherif M. Gohar, Ingrid Dieset, Nils Eiel Steen, Ragni H. Mørch, Trude S. Iversen, Vidar M. Steen, Ole A. Andreassen, Ingrid Melle

**Affiliations:** 1 K.G. Jebsen Centre for Psychosis Research, Institute of Clinical Medicine, NORMENT, University of Oslo, Oslo, Norway; 2 Psychosis Research Unit/TOP, Division of Mental Health and Addiction, Ullevål Hospital, Oslo University Hospital, Building 49, Kirkeveien 166, 0424 Oslo, Norway; 3 Department of Clinical Science, K.G. Jebsen Center for Psychosis Research, NORMENT, University of Bergen, Bergen, Norway; 4 Dr. Einar Martens Research Group for Biological Psychiatry, Center for Medical Genetics and Molecular Medicine, Haukeland University Hospital, Bergen, Norway; 5 Department of Psychiatry, Faculty of Medicine, Cairo University, Cairo, Egypt

**Keywords:** Depression, Dyslipidemia, Schizophrenia, Cholesterol, Cytokines, Bipolar

## Abstract

Although the relationship between positive and negative symptoms of psychosis and dyslipidemia has been thoroughly investigated in recent studies, the potential link between depression and lipid status is still under-investigated. We here examined the association between lipid levels and depressive symptomatology in patients with psychotic disorders, in addition to their possible inflammatory associations. Participants (*n* = 652) with the following distribution: schizophrenia, schizophreniform and schizoaffective disorder (schizophrenia group, *n* = 344); bipolar I, II, NOS, and psychosis NOS (non-schizophrenia group, *n* = 308) were recruited consecutively from the Norwegian Thematically Organized Psychosis (TOP) Study. Clinical data were obtained by Positive and Negative Syndrome Scale (PANSS), and Calgary Depression Scale for Schizophrenia (CDSS). Blood samples were analyzed for total cholesterol (TC), low-density lipoprotein (LDL), triglyceride (TG), C-reactive protein (CRP), soluble tumor necrosis factor receptor 1(sTNF-R1), osteoprotegerin (OPG), and interleukin 1 receptor antagonist (IL-1Ra). After adjusting for age, gender, BMI, smoking, and dyslipidemia-inducing antipsychotics, TC and LDL scores showed significant associations with depression [*β* = 0.13, *p* = 0.007; *β* = 0.14, *p* = 0.007], and with two inflammatory markers: CRP [*β* = 0.14, *p* = 0.007; *β* = 0.16, *p* = 0.007] and OPG [*β* = 0.14, *p* = 0.007; *β* = 0.11, *p* = 0.007]. Total model variance was 17% for both analyses [*F*(12, 433) = 8.42, *p* < 0.001; *F*(12, 433) = 8.64, *p* < 0.001]. Current findings highlight a potential independent role of depression and inflammatory markers, CRP and OPG in specific, in the pathophysiology of dyslipidemia in psychotic disorders.

## Introduction

Patients with schizophrenia and other psychotic disorders tend to have higher serum lipid levels than the general population [1]. The well-documented metabolic adverse effects of antipsychotics, together with unhealthy lifestyle that includes smoking and poor dietary habits, aggravate this phenomenon, affect the overall prognosis and could increase the risk of premature death in this group of patients [2, 3].

Alteration in lipid levels has been found not only in medicated, chronic patients, but also in drug-naive patients with first-episode psychosis, as demonstrated in a recent meta-analysis comprising 19 studies which revealed higher levels of triglycerides (TG) and lower levels of total cholesterol (TC) in patients compared to healthy controls [4]. The baseline dyslipidemia found in this study, in addition to the shared genetic overlap between schizophrenia and cardio-metabolic risk factors found in genome-wide association studies (GWASs), especially the loci related to triglyceride, low- and high-density lipoproteins cholesterol (mainly loci on gene regions: TCF4, TRIM26, MAD1L1 and MMP16) gives rise to the possibility of a disease-specific lipid pathway underlying the pathophysiology of psychotic disorders [5].

On the other hand, the relation between lipid disturbances and clinical characteristics of psychotic disorders is still not well understood, mainly due to fluctuation of symptoms severity as the disease progress and the presence of multiple factors that affect lipid levels. The findings of a recent 5-year follow-up study about the associations between higher lipids levels, severity of psychotic symptoms and level of functioning support the idea that raised lipid levels could be a disease trait in schizophrenia [6]. However, it is not clear if this association is specific to schizophrenia, and whether it is related to specific clusters of symptoms or symptom domain, i.e. only positive and negative symptoms or if it could be also related to depressive symptoms.

A relation between depression and lipid disturbances has been demonstrated in a substantial body of research [7–9], and depressed patients are also found to be at higher risk of developing cardiovascular diseases [10]. In addition, a recent study demonstrated that high levels of TC, LDL, TG, glucose and low level of HDL are present across schizophrenia, bipolar and unipolar mood disorders [11]. Another follow-up study of first-episode psychosis patients demonstrated an association between increased high-density lipoprotein (HDL) and reduced negative symptoms after 1 year of treatment with antipsychotic medications [12]. Although there are indications that dyslipidemia is associated with increased severity of psychotic symptoms, and possibly associated also with depressive symptoms in the spectrum of psychotic disorders, this has previously not been studied in detail.

In addition, large body of research suggests that inflammation and immune dysfunction might have a potential role in the pathophysiology of schizophrenia [13, 14], depression [15] and dyslipidemia [16]. Moreover, inflammation has been suggested to be the underlying mechanism that links depression and metabolic syndrome [17, 18]. In earlier study, a set of inflammatory cytokines hase been investigated in patients with psychotic spectrum disorders including interleukin 1 receptor antagonist (IL-1Ra), soluble tumor necrosis factor receptor 1 (sTNF-R1), osteoprotegerin (OPG), and von Willebrand factor (vWf) [13]. Being a soluble member of the superfamily of TNF-R, osteoprotegerin (OPG) is currently considered a potential marker that is involved in both cardiovascular diseases [19] and severe mental disorders by several hypothesized mechanisms mainly related to inflammation and calcium homeostasis [20]. There is thus an increased need to investigate its role in depression and lipid dysfunction within psychotic disorders.

The primary aim of the current study is to investigate the association between serum lipid levels (total cholesterol, LDL and triglycerides) and depressive symptoms in a large clinical sample of patients with psychotic disorders; more specifically to investigate if higher levels of serum lipids are associated with higher levels of depressive symptoms. A secondary aim is to assess the possible clinical and inflammatory associations to dyslipidemia.

## Methods

Subjects were recruited consecutively as part of the Thematically Organized Psychosis (TOP) Study between 2003 and 2015 from psychiatric departments of five major hospitals in the catchment areas of greater Oslo, Norway. The study was approved by the Regional Committee for Medical Research Ethics. After providing a written informed consent, participants were assessed with comprehensive clinical and biochemical assessments. Inclusion criteria for the TOP study are: age between 18 and 65 years and having a diagnosis of psychotic disorder within the schizophrenia and bipolar spectrum disorders according to DSM-IV. Participants with mental retardation, significant head injury, neurological disorder, or autoimmune disease were excluded from the study. More details about the TOP study are described in [21, 22].

### Participants

For the current study, we included subjects with one or more psychotic episodes and available serum lipid measurements. We excluded patients with CRP above 10 mg/L (*n* = 44) and patients with missing clinical, lipid or inflammatory data at time of recruitment, which are related to the main research question of the study. The final sample consisted of 652 participants with the following distribution of diagnoses: schizophrenia group (*n* = 344) which includes schizophrenia (*n* = 247), schizophreniform (*n* = 36) and schizoaffective disorder (*n* = 61); and non-schizophrenia group (*n* = 308) which includes bipolar I (*n* = 126), bipolar II (*n* = 58), bipolar NOS (*n* = 10), and psychosis NOS (*n* = 114).

### Procedures

#### Clinical variables

Demographic and clinical data were obtained by clinical interviews and from medical records. Diagnosis was established by use of the Structured Clinical Interview for DSM-IV Axis I Disorders (SCID-I) [23]. Current symptomatology was assessed by the following scales: the Structured Interview for the Positive and Negative Syndrome Scale (PANSS) [24]. The Calgary Depression Scale for Schizophrenia (CDSS) was used to assess depressive symptoms in patients with psychosis apart from negative and extrapyramidal symptoms [25]. We calculated duration of illness using the age at time of the study inclusion minus age of onset of first psychotic, manic, hypomanic or depressive episode. Standardized procedures were conducted in physical examination including height, weight and body mass index (BMI) together with a routine checkup by a physician to exclude any signs of ongoing infections.

#### Biochemical variables

Fasting venous blood samples were obtained in the morning. Serum lipids, i.e. total cholesterol (TC), triglyceride (TG) and low-density lipoproteins (LDL) were measured and analyzed according to standard techniques at the Department of Clinical Biochemistry, Oslo University Hospital. We added to the analysis a selected set of robust inflammatory markers, i.e. C-reactive protein (CRP), soluble tumor necrosis factor receptor 1 (sTNF-R1), osteoprotegerin (OPG) and interleukin-1 receptor antagonist (IL-1Ra) which have a potential role in psychosis pathophysiology [13, 26, 27].

#### Psychotropic medications

Pharmacological treatment may affect lipid levels as reported in many studies [28, 29]. We retrieved medication data from medical records, clinical interviews and measurements of antipsychotic drugs in serum of the participants to assess the degree of regularity of medications. Then, we categorized them into two subgroups according to their level of dyslipidemic effects (i.e. clozapine/olanzapine group vs other antipsychotics) [30].

### Statistical analysis

**Fig. 1:**
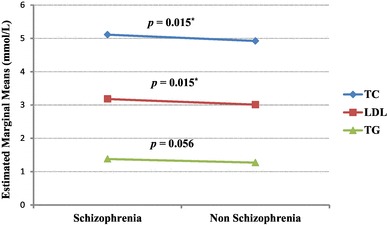
Marginal means in (mmol/L) of total cholesterol (TC), low-density lipoprotein (LDL) and triglyceride (TG) in schizophrenia and non-schizophrenia groups after adjusting for age, sex, BMI, smoking, duration of illness and the use of antipsychotics inducing dyslipidemia. *p < 0.05

The data were treated using the Statistical Package for Social Sciences (IBM SPSS Statistics v. 24) (SPSS Inc., Chicago, IL, USA/IBM, New York, USA). The level of statistical significance was preset to *p* < 0.05 (two tailed). Variables were presented as percentage and mean (± standard deviation) as appropriate. *t* test and the Chi-square test were used for comparisons between schizophrenia and non-schizophrenia groups regarding sociodemographic, clinical and biochemical variables (Table 1). One-way between-group analysis of covariance (ANCOVA) was added to assess the effect of putative confounders on serum lipid levels. Covariates were a priori selected on the basis of previously reported associations with lipids, in addition to variables that showed significant bivariate association with lipid levels in our sample analysis (Fig. 1).

**Table 1:** Subject characteristics in schizophrenia and non-schizophrenia groups

	Schizophrenia *n* = 344	Non-schizophrenia *n* = 308	Statistics
Age (years), *M* (SD)	28.67 (8.82)	31.33 (10.70)	*t* = − 3.44**
Female gender, *n* (%)	148 (43)	168 (54.5)	*χ*2 = 8.64**
Education (years), *M* (SD)	12.95 (2.82)	14.16 (2.87)	*t* = − 5.44**
Caucasian ethnicity, *n* (%)	269 (78.2)	261 (84.7)	*χ*2 = 4.57*
Daily smoking, *n* (%)	202 (58.7)	155 (50.3)	*χ*2 = 4.62*
Duration of illness (years), *M* (SD)	5.61 (6.63)	7.28 (8.56)	*t* = − 2.77**
Psychotropic medications			
Use on regular basis, *n* (%)	303 (88.1)	240 (77.9)	*χ*2 = 12.05**
Antipsychotics inducing dyslipidemia, *n* (%)	131 (38.1)	67 (21.8)	*χ*2 = 20.50**
PANSS positive, *M* (SD)	15.84 (5.41)	11.46 (4.09)	*t* = 11.73**
PANSS negative, *M* (SD)	16.34 (6.14)	11.32 (4.50)	*t* = 11.98**
CDSS, *M* (SD)	6.36 (4.45)	6.11 (4.43)	*t* = 0.73
BMI (kg/m^2^), *M* (SD)	25.54 (4.90)	25.47 (4.45)	*t* = 0.19
TC (mmol/L), *M* (SD)	5.10 (1.03)	4.93 (1.02)	*t* = 2.10*
LDL (mmol/L), *M* (SD)	3.18 (0.95)	2.99 (0.88)	*t* = 2.59**
TG (mmol/L), *M* (SD)	1.41 (0.87)	1.24 (0.65)	*t* = 2.90**
CRP (mg/L), *M* (SD) (*n* = 446)	3.04 (2.61)	2.79 (2.48)	*t* = 1.04
sTNF-R1 (ng/mL), *M* (SD) (*n* = 441)	1.88 (0.71)	1.85 (0.68)	*t* = 0.49
OPG (ng/mL), *M* (SD) (*n* = 446)	1.33 (0.40)	1.34 (0.42)	*t* = − 0.60
IL-1Ra (ng/mL), *M* (SD) (*n* = 446)	540.31 (1282.43)	445.07 (1156.56)	*t* = 0.82

*M* mean, *SD* standard deviation, *t* Student’s *t* test, *χ*^2^ Chi-square test, *PANSS* Positive and Negative Syndrome Scale, *CDSS* Calgary Depression Scale for Schizophrenia, *BMI* body mass index, *TC* total cholesterol, *LDL* low-density lipoprotein, *TG* triglyceride, *CRP* C-reactive protein, *sTNF*-R1 soluble tumor necrosis factor receptor 1, *OPG* osteoprotegerin, I*L-1Ra* interleukin 1 receptor antagonist

*p < 0.05, **p < 0.01

To investigate the association between symptom profiles, lipid levels and inflammatory markers, we explored their bivariate association using Pearson’s r for normally distributed continuous variables as shown in Table 2. To investigate the possible risk factors for hyperlipidemia, we conducted a hierarchal block-wise multiple linear regression analysis. TC and LDL were entered in two separate analyses as the dependent (outcome) variables with symptom profiles (positive, negative and depression) as independent variables in the first block. In the second block, we entered variables that showed associations with both TC and LDL in the bivariate analyses, i.e. age, gender, BMI, smoking, duration of illness, diagnostic categories and antipsychotics. In the third and final block, we entered inflammatory markers that showed significant correlation with TC and LDL, i.e. CRP and OPG. The residual plots showed satisfactory model fits and the analyses are shown in Table 3.

**Table 2:** Correlation between symptom profiles, serum lipid levels and inflammatory markers

	PANSS positive	PANSS negative	CDSS	TC	LDL	TG	CRP	OPG	sTNFR1
TC (*n* = 652)	0.05	0.07	0.11**	1					
LDL (*n* = 652)	0.08	0.10**	0.11**	0.92**	1				
TG (*n* = 652)	0.07	0.15**	0.04	0.37**	0.32**	1			
CRP (*n* = 446)	0.01	0.07	0.06	0.23**	0.26**	0.26**	1		
OPG (*n* = 446)	0.00	− 0.05	− 0.04	0.19**	0.13**	0.01	0.06	1	
sTNFR1 (*n* = 441)	0.002	0.02	− 0.004	− 0.02	0.03	0.10*	0.14**	0.11*	1
IL-1RA (*n* = 446)	− 0.01	− 0.04	0.04	− 0.02	− 0.02	− 0.02	− 0.02	0.10*	0.01

Bivariate associations were investigated using Pearson’s r correlation coefficient

*p < 0.05, **p < 0.01

*TC* total cholesterol, *LDL* low-density lipoprotein, *TG* triglyceride, *CRP* C-reactive protein, *sTNF*-*R1* soluble tumor necrosis factor receptor 1, *OPG* osteoprotegerin, *IL*-*1Ra* interleukin 1 receptor antagonist

**Table 3:** Multivariate regression analysis

	Serum TC levels			Serum LDL levels		
	Standardized beta coefficients			Standardized beta coefficients		
	Model 1	Model 2	Model 3	Model 1	Model 2	Model 3
PANSS positive	0.02	0.01	0.003	0.02	− 0.003	− 0.01
PANSS negative	0.05	0.05	0.04	0.08	0.05	0.05
CDSS	0.10*	0.13**	0.13**	0.10*	0.14**	0.14**
Age	–	0.34**	0.30**	–	0.28**	0.25**
Female gender	–	− 0.02	− 0.06	–	− 0.13**	− 0.16**
BMI	–	0.12**	0.07	–	0.17**	0.11*
Daily smoking	–	0.05	0.02	–	0.04	0.02
Duration of illness	–	− 0.06	− 0.05	–	− 0.08	− 0.07
Schizophrenia group	–	0.07	0.06	–	0.07	0.07
AP inducing dyslipidemia	–	0.12**	0.09	–	0.10*	0.07
CRP	–	–	0.14**	–	–	0.16**
OPG	–	–	0.14**	–	–	0.11*
	Adjusted *R*^2^ = 0.01 *F*(3, 442) = 2.10 *p* = 0.099	Adjusted *R*^2^ = 0.14 *F*(10, 435) = 8.04 *p* < 0.001	Adjusted *R*^2^ = 0.17 *F*(12, 433) = 8.42 *p* < 0.001	Adjusted *R*^2^ = 0.01 *F*(3, 442) = 3.18 *p* = 0.024	Adjusted *R*^2^ = 0.14 *F*(10, 435) = 8.31 *p* < 0.001	Adjusted *R*^2^ = 0.17 *F*(12, 433) = 8.64 *p* < 0.001

*TC* total cholesterol, *LDL* low-density lipoprotein, *PANSS* Positive and Negative Syndrome Scale, *CDSS* Calgary Depression Scale for Schizophrenia, *BMI* body mass index, *AP* antipsychotics, *CRP* C-reactive protein, *OPG* osteoprotegerin

*p < 0.05, **p < 0.01

## Results

Description of the participants and group comparison between the schizophrenia and non-schizophrenia groups are summarized in Table 1. The depression scores in the schizophrenia group were not statistically different from the non-schizophrenia group (6.36 ± 4.45 compared to 6.11 ± 4.43, respectively) (*t* = 0.73, *p* = 0.468). In addition, there was no statistically significant difference between the groups regarding BMI, with slightly higher values in schizophrenia (25.54 ± 4.90) than non-schizophrenia (25.47 ± 4.45). In contrast, the schizophrenia group had higher levels of TC (*t* = 2.10, *p* = 0.036), LDL (*t* = 2.59, *p* = 0.010) and TG (*t* = 2.90, *p* = 0.004) compared to non-schizophrenia. These higher levels remained significant for TC and LDL after adjustment for age, gender, BMI, smoking, duration of illness, and medications as shown in Fig. 1.

In the bivariate analyses, we found high levels of TC and LDL to be significantly associated with depressive symptoms (*r* = 0.11, *p* = 0.007; *r* = 0.11, *p* = 0.004), whereas high levels of TG and LDL were significantly correlated with negative symptoms (*r* = 0.15, *p* < 0.001; *r* = 0.10, *p* = 0.009). There were no significant bivariate correlations between lipid levels and positive symptoms. CRP showed significant positive correlation with lipid levels; TC (*r* = 0.23, *p* < 0.001), LDL (*r* = 0.26, *p* < 0.001) and TG (*r* = 0.26, *p* < 0.001). In addition, OPG was significantly correlated with TC (*r* = 0.19, *p* < 0.001) and LDL (*r* = 0.13, *p* = 0.005). While sTNFR1 showed significant positive association only with TG (*r* = 0.10, *p* = 0.040). Other correlations between lipid levels and inflammatory markers did not reach statistical significance (Table 2).

In the regression analyses, depression severity as measured by CDSS appeared to be the sole clinical symptom domain associated with high levels of both TC and LDL. After multivariate adjustment for age, gender, BMI, smoking, duration of illness, diagnostic categories and use of dyslipidemia-related antipsychotic medication (i.e. clozapine and olanzapine), the CDSS score remained significantly associated with high TC (*β* = 0.13, *p* = 0.007) and LDL (*β* = 0.14, *p* = 0.007) levels. Other significant associations of high LDL levels were higher age (*β* = 0.25, *p* < 0.001) and BMI (*β* = 0.11, *p* = 0.027), whereas female gender was associated with lower LDL levels (*β* = **−** 0.16, *p* = 0.001). Moreover, both CRP and OPG were found to be significantly associated with increased levels of TC and LDL. The total variance explained by the model for both TC and LDL analyses was 17% [*F*(12, 433) = 8.42, *p* < 0.001; *F*(12, 433) = 8.64, *p* < 0.001] as shown in Table 3.

## Discussion

We here demonstrate that high levels of TC and LDL were positively associated with depression in patients with psychotic disorders. This association remained significant after adjusting for potential confounding factors, including BMI, smoking status and use of dyslipidemia-related antipsychotic medications. Mean depressive symptoms scores in both the schizophrenia and non-schizophrenia groups were just around the cutoff score of 6 points of CDSS, which has been considered to identify depression in patients with schizophrenia [31]. Although depression means were not uniformly high, this does not exclude the potential negative impact of depression even on subsyndromal level on quality of life, level of functioning, degree of distress and medical comorbidity as reported in several studies [32–34].

To the best of our knowledge, this is the first study that has investigated the role of depression in particular in association with dyslipidemia in patients with schizophrenia and other psychotic disorders. Dyslipidemia represents an important component of metabolic syndrome according to the National Cholesterol Education Program’s Adult Treatment Panel III report (ATP III) [35]. Previous studies have addressed the significant association between depression and metabolic syndrome in general [36–38], and in schizophrenia [39]. It is important to note that the relationship between depression and metabolic syndrome could be bidirectional and reciprocal, as demonstrated by a meta-analysis conducted on both cohort and cross-sectional studies [40]. The same pattern of association was present with depression in relation to diabetes [41] and obesity [42]. This pattern could be extended to dyslipidemia, especially in psychotic disorders.

One of the major pathophysiological mechanisms possibly underlying the interplay between depression and dyslipidemia within the psychotic spectrum is the inflammatory pathway. Depression and schizophrenia have been firmly shown to be associated with elevated levels of several inflammatory cytokines [15, 43]. In addition, patients with metabolic syndrome tend to have increased levels of cytokines [44], which may also be involved in associated depression [45]. The positive correlation between CRP, OPG and lipid levels in our sample supports the potential role of inflammatory pathway. The association between lipids and OPG has been demonstrated in many studies. For example, an animal study concluded that dyslipidemia resulted in increased OPG expression in mice [46]. While in humans, OPG levels were positively correlated with serum TC and LDL in a sample of 286 healthy women [47]. Moreover, it was reported that the use of statin therapy resulted in the reduction of OPG levels in patients with coronary artery disease [48].

It is worth noting that we did not find associations between levels of depression and any of the inflammatory markers included in this study. Similar results have been reported previously in a sub-sample of the present study, which found associations between sTNF-R1, IL-1Ra, OPG, IL-6 and severity of mood symptoms only in patients with bipolar disorders, but not in schizophrenia [49]. Although depression is associated with elevated levels of inflammatory markers in many studies [15], depressive symptoms in schizophrenia may have a specific profile and a different pathophysiology compared to major depressive and other affective disorders [50]. This specific profile could be associated with different immune profiles. Further, it is still not clear if increased level of cytokines represents a state or trait marker, especially since this study sample had depression severity means just around the cutoff score of CDSS.

Limitations of this study include the cross-sectional design and the inability to rule out other unknown confounders that could have influenced our findings, in particular, that are related to lipid levels (e.g. dietary habits and exercise). It is worth to mention that we adjusted as much as possible for potential confounding factors such as medications, but this could not abolish the potential effect of other antipsychotics on lipid metabolism by direct and indirect mechanisms, e.g. hyperprolactinemia and weight gain documented with first-generation antipsychotics [51]. In addition, the measurements of lipid and inflammatory markers in peripheral blood may not necessarily reflect what is present in the brain. However, evidence from animal studies indicates potential communication across the blood–brain barrier. For example, synthesis of brain cholesterol has been reduced in guinea pigs under the effects of cholesterol-lowering statins [52]. Within the same context, studies on inflammatory cytokines have shown strong correlation between markers in peripheral blood and CSF [53]. Finally, we did not include the healthy controls in the association study since controls with mental symptoms were screened out.

In conclusion, findings from the current study tried to highlight the possible contributing role of depression in dyslipidemia within psychotic disorders together with the potential role of inflammatory markers in the pathophysiology of this important component of metabolic syndrome. Being an observational study, we cannot prove causality, but we can raise potential hypotheses for further investigation. Further studies are required to understand the interaction between inflammatory pathways and lipids on the one side, and their relation to the clinical profile of psychotic disorders on the other side, with particular emphasis on the role of depressive symptomatology. Exploring such complex mechanisms may be crucial for prevention and treatment of both dyslipidemia and depression within the psychotic spectrum.
